# Medical History from the Earliest Times

**Published:** 1893-01-28

**Authors:** E. T. Withington


### MEDICAL HISTORY FROM THE EARLIEST TIMES.

By E. T. WITHINGTON, M.A., M.B. Oxon.

### XL.—THE CLOSE OF THE MIDDLE AGES.

THE interval between the end of the thirteenth century and the beginning of the Renaissance (1300—1450) is marked by many signs of the approaching dawn, but we must here confine ourselves to consider the work of Mondino of Bologna, "restorer of anatomy," Guy of Chauliac, "restorer of surgery," and the chief writers of those "consilia," or consultations, which form the characteristic medical literature of the period.

In contrast to the ancient Greeks, the early Christians looked upon their living bodies with contempt, but they regarded them after death with even more than Greek reverence, for was not every particle destined to be raised in glory? Human anatomy was, therefore, out of the question, and even the dissection of animals might bring upon the surgeon an accusation of sorcery or of attempting to restore the pagan arts of divination. We have seen, however, that the students of Salerno studied the anatomy of the pig as early as the eleventh century, and in the thirteenth the Emperor Frederick II. ordered that a human body should be dissected at least once in five years. Some scanty knowledge might also be obtained from another source contrasting strangely with those now employed. During the Crusades princes and nobles were wont to take with them, as part of their camp furniture, a large cauldron, in which, in case of death, their bodies were boiled, and the bones extracted and brought back to their fatherland. So was it done to many Bishops and Knights who accompanied Frederick Barbarossa to Italy in 1167, and to that Emperor himself when he was drowned in the river Saleph. St. Louis, of France, Philip the Bold, and his Queen, were treated in the same way till, in A.D. 1300, the practice was entirely prohibited by a bull of Pope Boniface VIII. As already indicated, post-mortem examinations were by no means unknown in the thirteenth century, and human anatomy was probably studied more frequently than is generally supposed. It was certainly practised by Peter of Abano, and Peter of Abano was a friend of Mundinus or Mondino de Luzzi. The latter became professor at Bologna about 1290, and in 1316 he published his "Anathomia," the first work since the days of the Alexandrine anatomists founded on actual dissection of human subjects, in which he is said to have been assisted by a learned lady, Alexandra Giliani.

Bologna now became the foremost of medical schools, and among the students attracted thither by the fame of Mondino and his pupils was Guy of Chauliac, who also studied at Montpellier and Paris, and afterwards became surgeon and "caplanus commensalis" to three Popes, Clement VI., Innocent VI., and Urban V. (1352-78). Guy holds the same position in surgery as Mondino does in anatomy, both are said to have "restored" their respective arts, that is their works possessed sufficient originality and excellence to take the place of translations from Galen and Albucasis, but the creation of a new anatomy and a new surgery was reserved for their greater successors, Vesalius and Ambrose Paré.

Guy of Chauliac was not only the ablest practitioner, but also the most cultured surgeon of his age. He reverences the Greeks and Arabs, especially "our father Galen," and the great Albucasis, and he admits that his work on surgery is mainly compiled from their writings, "with the addition of a few things which, according to my moderate intelligence, seemed to be useful. For we are like children on the neck of a giant, who see all the giant sees and something besides." His contemporaries, he says, "follow one another like cranes, and despise everything not sanctioned by custom and authority, forgetting that Aristotle declares, in the second book of his metaphysics, that these are the two great hindrances to the discovery of truth." But most of them know nothing of Aristotle, and both surgeons and physicians so neglect their general education, that, if they persist in doing so, he will not be surprised to see tanners and carpenters deserting their trades and taking to physic. A good surgeon should be acquainted with liberal studies, with medicine, and, above all, with anatomy; "he should be courteous and condescending, bold in security, cautious in time of danger, avoiding impracticabilities, compassionate to the infirm, benevolent to his associates, circumspect in prognosis, chaste, sober, pious, and merciful, not greedy of gain, no extortioner, but looking for his fee in moderation, according to the extent of his services, the ability of his patient, the result of his treatment, and a proper sense of his own dignity." The surgeons of the preceding century had been little more than wound curers, and, as we have seen, were divided into sects according to their various modes of dealing with such injuries; but in Guy's work other branches of the art, and especially the treatment of fractures and dislocations, which was comprised under the curious term "Algebra," first became prominent, and we find many practices recommended which are generally supposed to have been of much later origin. Thus, he advises that bandages should be stiffened by being dipped in white of egg; he suspends fractured limbs in a sort of cradle, "cunabulum aut suspensorium"; and he treats fractures of the thigh not only by long splints, but also by the pulley and weight, "ad pedem ligo pondus plumbi transeundo chordam super parvam polegeam." One of the most striking objects of the hospital ward seems to have originated with him, for he recommends that a rope should be suspended over the patient's bed to assist him in lifting or turning himself.

The philosopher Seneca notices with just censure a custom among the physicians of his day of writing letters of advice to patients they had never seen. The habit is not yet extinct, but it formed only one, and that the least important, source of the medical "consilia" of the fourteenth and fifteenth centuries. Others consist of extracts from the case books of distinguished physicians, published for the benefit of their colleagues and successors, while a third class comprises answers to inquiries sent by country practitioners or former pupils to celebrated professors, who thus became the prototypes of the modern consultant. The "consilia" are far superior in interest and originality to the old compilations, and mark an important step towards the reproduction of the clinical observations of Hippocrates. The chief writers were Italians: Gentilis of Foligno (1348), Antony Cermisone (1443), and Bartholomew Montagnana (1470), all three professors at Padua, but we must confine ourselves to one example from the last-named author. A typical consilium begins with a brief description of the patient, his temperament, and his disease; it then suggests what may be the causes of this, and warns him of the dangers he incurs if he does not follow the advice given under the heads of regimen, diet, and drugs respectively. The following is the shortest of the 305 consilia of Bartholomew Montagnana: "A lady with an ulcer in her ear, discharge of pus, noises, and decrease of hearing. There is great fear that this will result in complete deafness, which may be prevented thus: Let her avoid over-eating, and refrain from pastry, milk, and all milk foods, from boiled fish, especially tench and eels, and from vegetables and stewed meats, above all at night. She must avoid sweet wines, and must add one-fourth water to what she does drink, refrain from active exercise after food, and must not hang her head down. As to medical treatment: (1) Let her be bled to four or five ounces from the right cephalic vein, or that between the thumb and index of the right hand; (2) a rhubarb pill in the morning; (3) a poultice, chiefly of powdered asarum root, to be applied locally for about half-an-hour daily; (4) an oil, two or three drops of which are to be run into the ear four times a day; (5) another purgative pill at bed time; (6) some drops of cyclamen juice to be used for ten days alternately with the above oil. By so continuing the patient will be cured *ad laudem Dei Omnipotentis*. Amen."

